# Targeting the cGAS-STING pathway: emerging strategies and challenges for the treatment of inflammatory skin diseases

**DOI:** 10.3389/fphar.2025.1597443

**Published:** 2025-06-09

**Authors:** Haoyun Luo, Tian Tian, Chenmin Hu, Fei Hao

**Affiliations:** Dermatology and Plastic Surgery Center, The Third Affiliated Hospital of Chongqing Medical University, Chongqing, China

**Keywords:** cGAS-STING pathway, inflammatory skin diseases, immune response, inhibitor, combination therapy

## Abstract

The cGAS-STING signaling pathway is a major component of innate immunity. It is critical for identifying cytoplasmic DNA, triggering immune responses, and is linked to several diseases and malignancies. The inflammatory infiltrates and elevated cytokine expression characteristic of dermatological inflammatory disorders have drawn attention to the potential role of the cGAS-STING pathway, positioning it as an emerging focus of scientific investigation. Therapeutic strategies targeting the cGAS-STING signaling axis have been explored for the treatment of inflammatory dermatoses, with several inhibitor classes under investigation, such as cGAS inhibitors, STING palmitoylation blockers, STING trafficking suppressors, and nanoformulated inhibitors. Nevertheless, there are problems in both this pathway and drug research still left to be solved: inhibitors are not generally specific enough, drugs are not generally bioavailable enough, and there is an imbalance between the two—immunosuppression and the immune response. The several possible approaches in the future directions include combination therapy, multi-omics integration, and precision medicine approaches to treat these diseases. Given its broad immunomodulatory effects, there is high potential for clinical application of the cGAS-STING pathway modulators.

## 1 Introduction

The cyclic guanosine monophosphate-adenosine monophosphate synthase-stimulator of interferon genes (cGAS-STING) pathway controls the innate immune response and is a sentinel of self or foreign DNA in the cytoplasm (Ablasser and Chen, 2019). Upon recognition of double-stranded DNA, cGAS undergoes a conformational change and, forming the second messenger cyclic GMP-AMP (cGAMP). Subsequently, cGAMP binds to and activates STING (Ablasser and Chen, 2019). The activation of STING triggers a signaling cascade that leads to the production of type I interferons and pro-inflammatory cytokines (Wan et al., 2020). The cGAS-STING pathway has been implicated in both immune defense and pathological inflammation in several inflammatory diseases and cancers (Hao, 2023). Strict regulation of cGAS-STING signaling is required for maintaining the balance of an immune response.

Inflammatory skin diseases are characterized by a spectrum of cutaneous disorders accompanied by intense inflammatory cell infiltration and elevated levels of inflammatory cytokines (Morizane et al., 2023). Within the context of immune regulation and inflammatory processes, investigation of the cGAS-STING pathway has emerged as a focal point in current research across multiple disease states. Studies have revealed that tumor necrosis factor (TNF) and H_2_O_2_ facilitated the migration of nuclear DNA into the cytoplasm of keratinocytes, thereby enabling double-stranded DNA-mediated STING protein to evade degradation, this process triggers an inflammatory cascade in macrophages and keratinocytes of psoriasis (Yu et al., 2022). In systemic lupus erythematosus (SLE), activation of the cGAS-STING pathway exacerbates disease phenotype through multiple mechanisms, including induction of hyperactivation of the complement system, and production of inflammatory molecules such as type I interferons (IFN-α and IFN-β), TNF-α, and interleukins (IL-6 and IL-1) (Feng et al., 2024; Troldborg et al., 2018).

To facilitate therapeutic advancements targeting the cGAS-STING signaling pathway and its role in inflammatory skin diseases, it is imperative to comprehend the complexity of this signaling mechanism. We have systematically reviewed how the cGAS-STING pathway is activated in common chronic inflammatory skin conditions and discussed the potential applications of cGAS-STING signaling in treating cutaneous inflammatory disorders, along with the benefits and challenges in this emerging field of research (Decout et al., 2021).

## 2 cGAS-STING plays an important role in innate and adaptive immunity

Pathogenic infection, endogenous DNA damage, and DNA from tumor cells are the three major stimuli of the cGAS-STING signaling cascade. This pathway serves multiple critical biological functions, including antimicrobial defense, tumor immunosurveillance, regulation of cellular aging, autophagic processes, and modulation of autoimmune and inflammatory responses. The pathway comprises several essential components:

The cytoplasmic DNA sensor cGAS is a member of the highly conserved cGAS/DncV-like nucleotidyltransferase (CD-NTase) superfamily (Kranzusch et al., 2013). Comprised of N-terminal and C-terminal catalytic domains (Kranzusch et al., 2013; Kato et al., 2013), cGAS recognizes double-stranded DNA (dsDNA) via positively charged surface regions without sequence preference (Luecke et al., 2017). This recognition event induces conformational changes in cGAS, reconfiguring its catalytic site to enable synthesis of cGAMP from ATP and GTP substrates (Ablasser et al., 2013; Wu et al., 2013).

STING exists as a transmembrane protein anchored in the endoplasmic reticulum (ER). Its structural organization features N-terminal transmembrane helices (TM1-4) that anchor it within the ER membrane. In its inactive state, STING exists as a dimer (Kato et al., 2013). This results in structural changes in STING that induce oligomerization and translocation from the ER into the trans-Golgi network (Li et al., 2013). The cyclic dinucleotides (CDNs) bind to STING ligand-binding domain (LBD) and activate this dome structure to initiate higher-order oligomer assembly (Kato et al., 2013). The activation of STING complexes then recruits and activates TANK-binding kinase 1 (TBK1) (Parvatiyar et al., 2018). TBK1 subsequently phosphorylates interferon regulatory factor 3 (IRF3), which dimerizes and translocates to the nucleus, where it activates the expression of IFN- and other interferon-stimulated genes (ISGs). This promotes pathogen clearance by inhibiting replication and enhancing immune cell activity (Lama et al., 2019). A related outcome of this pathway is that it leads to the production of inflammatory molecules (TNF and IL-6), which create local or systemic inflammatory environments that facilitate pathogen elimination (Bennion et al., 2019; Hu et al., 2019; Su et al., 2019).

In order to prevent overactivation and maintain immune homeostasis, the cGAS-STING pathway is strictly regulated by a variety of negative feedback mechanisms.

### 2.1 Synergy with DNA repair machinery

DNA repair enzymes play critical roles in maintaining cellular homeostasis, with TREX1 (three prime repair exonuclease 1) and RNase H2 (ribonuclease H2) serving as two essential nucleases that prevent aberrant activation of the cGAS-STING signaling pathway through cytoplasmic DNA clearance (Simpson et al., 2020; Hiller et al., 2012). TREX1 functions as a potent 3′-5′ DNA exonuclease that specifically degrades cytosolic DNA to prevent inappropriate immune activation, and its dysfunction has been strongly associated with various autoimmune disorders (Simpson et al., 2020). RNase H2 primarily removes ribonucleotides misincorporated into DNA to preserve genomic integrity, and its deficiency leads to chronic activation of DNA damage response pathways (Hiller et al., 2012). Together, these enzymes constitute a vital surveillance system that safeguards against uncontrolled innate immune activation while maintaining proper nucleic acid metabolism.

### 2.2 Post-translational modification (PTM)

The post-transcriptional regulation of the cGAS-STING pathway precisely modulates immune responses by controlling mRNA stability, splicing, nuclear export, and translation. The RNA-binding protein LUC7 Like 2, Pre-MRNA Splicing Factor (LUC7L2) mediates intron retention in STING precursor mRNA, inhibiting its proper splicing and promoting mRNA degradation, thereby reducing STING protein levels and preventing excessive activation of antiviral immune responses (Li et al., 2021). Ubiquitination, as a crucial post-translational modification (PTM), plays a pivotal role in regulating STING (Stimulator of Interferon Genes) protein stability through the action of specific E3 ubiquitin ligases including RNF5 (Ring Finger Protein 5), TRIM29 (Tripartite Motif Containing 29), and TRIM30α (Tripartite Motif Containing 30α), which mediate its degradation to maintain immune homeostasis. RNF5 promotes K48-linked polyubiquitination of STING, targeting it for proteasomal degradation and suppressing STING-mediated interferon signaling (Yan et al., 2023), while TRIM29 negatively regulates innate immune responses to cytosolic DNA and DNA viruses by inducing K48-linked ubiquitination and degradation of STING, as evidenced by impaired STING degradation and enhanced STING-TBK1-IRF3 signaling in TRIM29 knockout cells (Li et al., 2018). Additionally, TRIM30α facilitates STING degradation through proteasome-dependent K48-linked ubiquitination at Lys275 (Wang et al., 2015), collectively demonstrating how these E3 ligases form a sophisticated ubiquitination network that precisely controls STING protein stability and activity to ensure proper termination of STING signaling and prevent excessive inflammatory responses. This aspect has been comprehensively reviewed by Chen Y et al., and therefore will not be elaborated in detail in the present review (Chen et al., 2025).

The negative feedback regulation of STING signaling involves additional molecular players including RIG-I (retinoic acid-inducible gene I) and IL-6 (interleukin-6), which contribute to maintaining immune homeostasis. RIG-I, a pattern recognition receptor that detects dsRNA, plays a dual role in both initiating antiviral responses through TBK1 and IκB kinase ε (IKKε)-mediated type I IFN production and subsequently terminating STING signaling (Kato et al., 2008). These molecules facilitate STING degradation through activation/dephosphorylation of UNC-51-like kinase 1 (ULK1), thereby limiting excessive innate immune responses triggered by cytosolic DNA and preventing potential autoimmune reactions (Wu et al., 2017).

This sophisticated regulatory network enables the immune system to mount effective responses against pathogens while maintaining self-tolerance and preventing tissue damage.

Apart from its main function in type I interferon signalling, the cGAS-STING pathway is involved in a multitude of non-canonical signalling pathways contributing to cellular and tissue homeostasis, as well as pathology. Among these is the cGAS-STING-PERK-eIF2 axis that affects cellular aging, organ fibrosis, and the regulation of the autophagy by STING, as well as the activation of the NFκB by STING, all resulting in inflammatory conditions, autoimmune disorders, and tumour immunity (Chen and Xu, 2023).

The cGAS-STING pathway had profoundly immunological effects as a critical mediator between innate and adaptive immunity. STING works in a variety of cell types including macrophages, DCs, lymphocytes, endothelial cells, and epithelial cells (Barber, 2015). It is obvious that this pathway plays a huge role in macrophage polarisation dynamics, a vital part of innate immunity and the basis of the potential of therapeutic intervention (Ni et al., 2023; Ou et al., 2021). In B cells, STING activation facilitates the degradation of membrane-bound immunoglobulin M (IgM), Igα, and Igβ via the SEL1L/HRD1-mediated endoplasmic reticulum-associated degradation (ERAD) pathway, thereby attenuating B cell receptor (BCR) signaling (Tang et al., 2021). This regulatory function is further corroborated by observations in STING V154M mutant mice, which exhibit a significantly diminished immune response to T cell-independent antigens, a marked reduction in antigen-specific plasma cells post-immunization, and decreased antibody production (Tang et al., 2021). Collectively, these findings underscore the essential role of STING in modulating BCR signaling and humoral immune responses within normal B-cell physiology.

**FIGURE 1:**
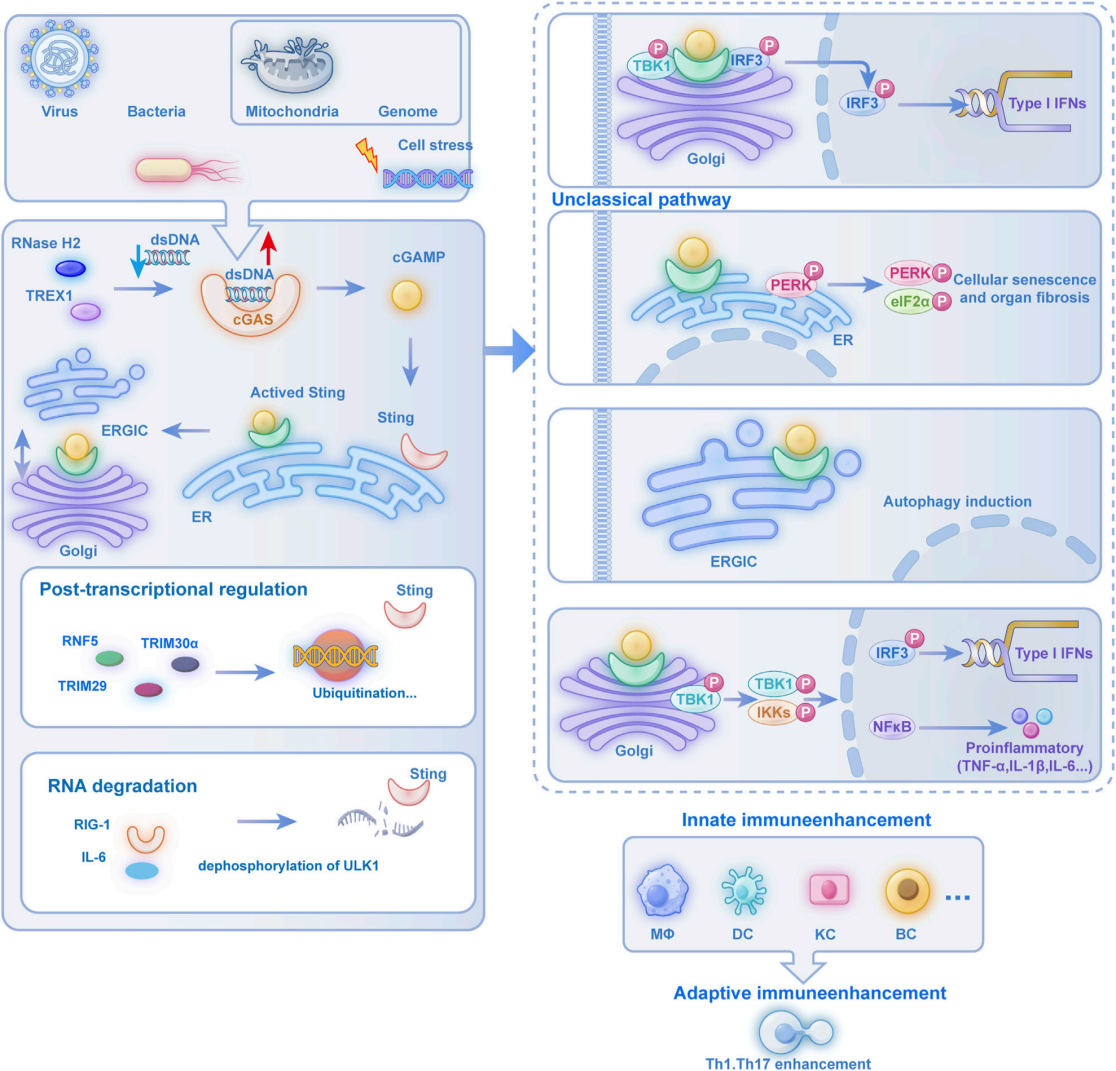
The Multifunctional Regulatory Role of the cGAS-STING Pathway in Immune and Cellular Pathophysiological Processes: cells activate the cGAS-STING pathway when encountering viral, bacterial, mitochondrial, or genomic DNA stress. cGAS recognizes dsDNA to produce cGAMP, which activates the ER-resident STING protein. Activated STING translocates through the ERGIC to the Golgi apparatus, where it recruits TBK1 to phosphorylate IRF3, leading to nuclear translocation and subsequent production of type I IFNs and proinflammatory cytokines, thereby enhancing innate immunity in MΦs, DCs, and KCs while bridging adaptive immunity through Th1 and Th17 cell activation. Beyond this classical pathway, STING activation also initiates non-canonical signaling cascades: at the Golgi, it triggers PERK-eIF2α signaling to drive cellular senescence and organ fibrosis; within the ERGIC, it induces autophagy; and through Golgi-localized TBK1 and IKK complexes, it activates both IRF3-dependent IFN-I production and NF-κB-mediated expression of proinflammatory cytokines such as TNF-α and IL-1β, demonstrating the pathway’s diverse functional repertoire in coordinating immune and cellular stress responses. cGAS-STING: cyclic GMP-AMP synthase–stimulator of interferon genes. Post-translational regulation involves ubiquitination by E3 ligases (RNF5, TRIM30α, TRIM29), with additional crosstalk through RIG-I and IL-6 signaling. ULK1-mediated dephosphorylation further modulates RNA degradation processes, demonstrating the pathway’s multifaceted immunoregulatory roles. Abbreviation Legend: cGAS-STING: cyclic GMP-AMP synthase–stimulator of interferon genes; dsDNA: cytosolic double-stranded DNA; cGAMP: cyclic GMP-AMP; ER: endoplasmic reticulum; ERGIC: ER-Golgi intermediate compartment; TBK1: TANK-binding kinase 1; IRF3: interferon regulatory factor 3; IFN-I: type I interferons; MΦs: macrophages; DCs: dendritic cells; KCs: keratinocytes; Th: T helper; PERK: protein kinase R-like endoplasmic reticulum kinase; eIF2α: eukaryotic initiation factor 2α; IKK: IκB kinase; NF-κB: nuclear factor kappa B; TNF-α: tumor necrosis factor-alpha; IL-1β: interleukin-1 beta; RNF5 (Ring Finger Protein 5); TRIM30α (Tripartite Motif Containing 30 Alpha); TRIM29 (Tripartite Motif Containing 29); RIG-I (Retinoic Acid-Inducible Gene I); IL-6 (Interleukin 6); ULK1 (Unc-51 Like Autophagy Activating Kinase 1).

Furthermore, cGAS-STING activation within antigen-presenting cells (APCs) during cellular stress initiates a cytokine cascade that orchestrates T lymphocyte recruitment, maturation, activation, and differentiation (Zhao et al., 2019), thereby coordinating subsequent adaptive immune responses (Li et al., 2019; Li and Chen, 2018) (Figure 1).

## 3 Abnormal activation of cGAS-STING and inflammatory skin diseases

**FIGURE 2:**
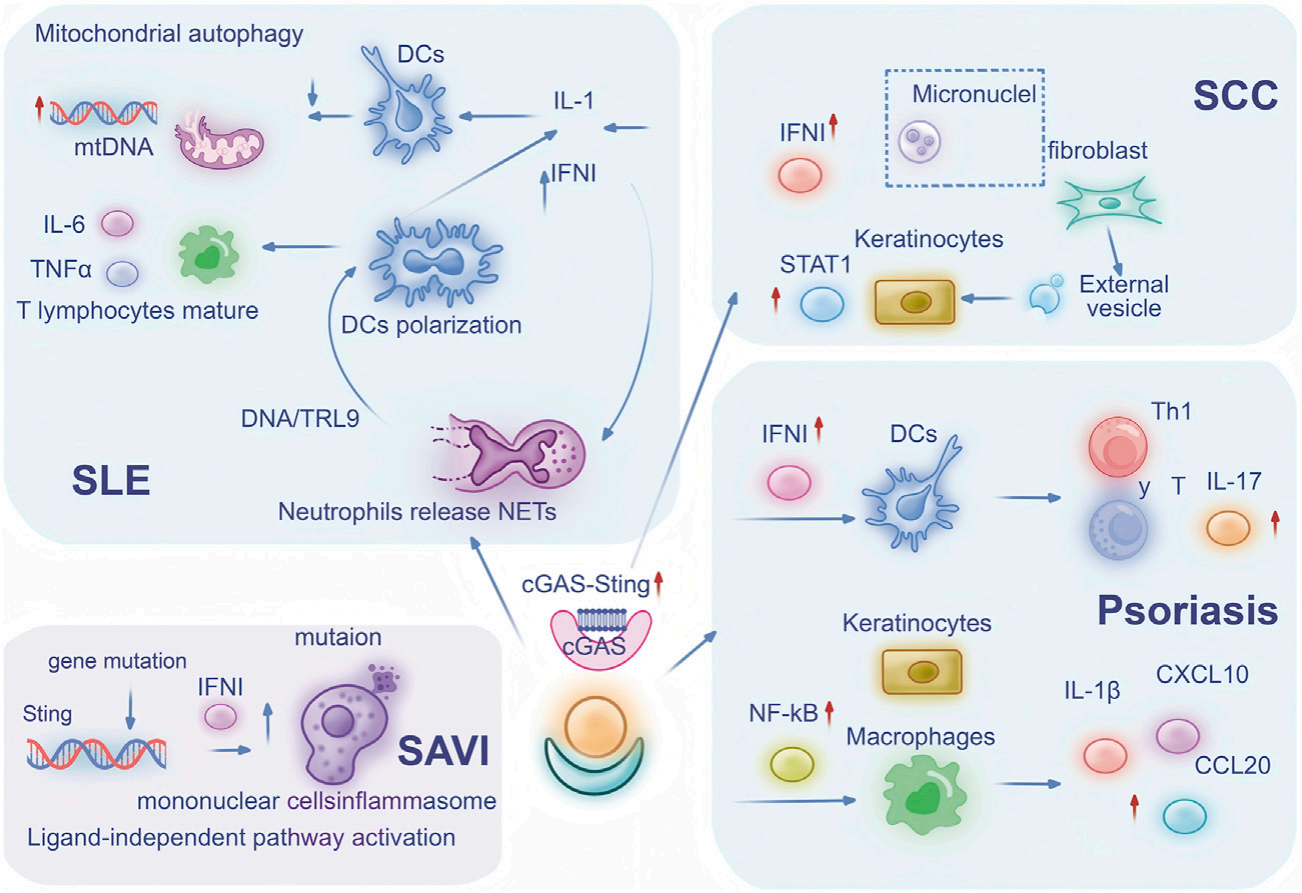
cGAS-STING Participates in Inflammatory Skin Diseases through Multiple Pathways: The central role of the cGAS-STING pathway in mediating immune cell signaling and inflammatory responses across four distinct diseases: SLE, SAVI, SCC, and psoriasis. In SLE, defective mitophagy leads to mtDNA release, triggering IL-6 and TNF-α secretion while neutrophil-derived NETs activate DCs through DNA/TLR9 pathways, resulting in polarized DCs producing IL-1 and IFN-I. SAVI features constitutive STING activation through genetic locus mutation, causing monocyte inflammasome activation and IFN-I upregulation. SCC pathogenesis involves fibroblast-derived micronuclei transferring to keratinocytes via extracellular vesicles, subsequently activating STAT1 and IFN-I signaling. Psoriasis exhibits IFN-I-stimulated DCs promoting Th1 and γδ T cell responses with IL-17 secretion, while keratinocyte-macrophage crosstalk activates NF-κB to induce IL-1β, CXCL10 and CCL20 production. Collectively, this integrated mechanism highlights how cell-cell interactions, cytokine networks, and signaling cascades converge through the cGAS-STING axis to drive diverse immunopathological processes across these conditions. Abbreviation Legend: cGAS-STING: cyclic GMP-AMP synthase-stimulator of interferon genes; SLE: systemic lupus erythematosus; SAVI: STING-associated vasculopathy with onset in infancy; SCC: cutaneous squamous cell carcinoma; mtDNA: mitochondrial DNA; IL-6: interleukin-6; TNF-α: tumor necrosis factor-alpha; NETs: neutrophil extracellular traps; DCs: dendritic cells; TLR9: Toll-like receptor 9; IL-1: interleukin-1; IFN-I: type I interferons; STAT1: signal transducer and activator of transcription 1; Th1: T helper 1; γδ T cell: gamma delta T cell; IL-17: interleukin-17; NF-κB: nuclear factor kappa-light-chain-enhancer of activated B cells; IL-1β: interleukin-1 beta; CXCL10: C-X-C motif chemokine ligand 10; CCL20: C-C motif chemokine ligand 20.

While acute inflammatory responses play an essential role in pathogen defense, persistent inflammation can trigger the onset of inflammatory skin disorders. Research has identified the cGAS-STING pathway as a central mediator of both immediate and sustained low-intensity inflammatory processes linked to various pathological conditions (Ablasser and Chen, 2019; McCaffary, 2017). Perturbations in cellular trafficking mechanisms can significantly alter STING functionality, resulting in inappropriate immune system activation. The following sections explore the pathway’s significance in prevalent inflammatory skin conditions. Pathological activation of cGAS-STING signaling contributes to numerous inflammatory and autoimmune disorders, which can be classified into two distinct categories based on their initiating mechanisms. The first involves excessive DNA accumulation, resulting in sustained cGAS-STING activation and continuous inflammatory cytokine production, particularly IFN-I. The second stems from STING mutations that bypass normal regulatory controls, leading to constitutive activation and subsequent autoimmune manifestations (Figure 2).

### 3.1 Systemic lupus erythematosus

Given the established role of type I interferons and self-nucleic acids as primary drivers in systemic autoimmune pathogenesis, the involvement of the cGAS-STING pathway in these disorders has garnered significant research attention, with this process encompassing complex regulatory mechanisms at multiple levels.

Research by An and colleagues revealed increased cGAS expression and elevated cGAMP concentrations in patients’ peripheral blood mononuclear cells (PBMCs), showing positive correlation with anti-dsDNA antibody levels and SLE disease activity index (SLEDAI) measurements (An et al., 2017). Additional investigations have shown that cGAS-STING activation enhances type I interferon production in SLE patient serum samples (Kato et al., 2018; Ding et al., 2015). Notably, aberrant mitochondrial DNA (mtDNA) release may serve as a critical trigger for cGAS-STING pathway activation. Several SLE phenotypes have been mimicked when the THP1 cell line was transfected with mitochondrial DNA (mtDNA) (Qin, 2012) Qin Z et al. demonstrated that mtDNA transfection not only induces type I interferon responses but also enhances glycolytic flux in THP1 cells, leading to increased lactate production (Zhang J. et al., 2024). In more precise terms, acetylation discouraged cGAS from interacting with E3 ubiquitin ligase, Membrane Associated Ring-CH-Type Finger 5 (MARCHF5), cleared for cGAS degradation, and then ensued a strong IFN-I response (Zhang J. et al., 2024).

Gao, D et al. demonstrated that cGAS deletion substantially reduced autoantibody generation and activated T cell populations in rex1-deficient mice (Gao et al., 2015). These observations highlight the synergistic interaction of immune cells in the pathologic course of SLE to amplify inflammation. This enhanced activation operates in a complex immune network involving multiple innate immune components, particularly plasmacytoid dendritic cells (pDCs) (Ganguly et al., 2013). These specialized innate immune cells demonstrate the capacity to internalize and detect self-DNA, triggering IFN-I production in SLE patients (Bode et al., 2016). Studies show that interferon α/β and IL-1 suppress mitochondrial autophagy in dendritic cells, impeding mitochondrial DNA elimination through cGAS-STING signaling (Aarreberg et al., 2019). Concurrently, monocytes utilize the cGAS-STING pathway to detect mitochondrial DNA, facilitating their differentiation into dendritic cells (Blanco et al., 2001). Additionally, neutrophil extracellular traps (NETs), activated by interferon α/β, contribute to pDCs stimulation (Lood et al., 2016). In the SLE context, IFNs prime mature neutrophils *in vivo*, triggering NET release that activates pDCs through DNA- and TLR9-dependent mechanisms, resulting in enhanced IFN-α production (Garcia-Romo et al., 2011). The above processes constitute a key bridge for the transition from innate immune system to adaptive immunitm, promoting T lymphocyte maturation, while elevated levels of pro-inflammatory mediators, particularly IL-6 and TNF-α, which provides a second signal for T cell activation by upregulating the expression of co-stimulatory molecules trigger adaptive immune responses (Wan et al., 2020). CD4+T cells play a central role in this immune cascade. The occurrence (de Andrés et al., 2007) and development of SLE are closely related to it (Li et al., 2022). Their ability to activate B cells to produce antibodies and promote inflammation is overactivated in SLE, leading to an aggravated autoimmune response (Li et al., 2022). Research by et al. using the SLE-prone *Fcgr2b* ^−/−^ mouse model revealed direct molecular interactions between cGAS, STING, and TBK1, enhancing IFN-I signaling and CD^4+^ T cell activation (Huang et al., 2023).

Despite ongoing debate regarding STING’s precise role in SLE pathogenesis due to disease heterogeneity, certain studies indicate that its absence may intensify disease manifestations. Research by Sharma S et al. revealed that mice with combined STING/Fas deficiency and autoimmune predisposition showed decreased expression of macrophage immune activation suppressors, resulting in enhanced TLR ligand responsiveness (Sharma et al., 2015). Additionally, Motwani M et al. demonstrated that cGAS and STING deficiency enhanced SLE autoantibody production and proteinuria levels in Pristane-induced lupus models. Further investigation using cGAS knockout Faslpr mice generated on pure MRL/Faslpr background through Crispr/Cas9 revealed the cGAS-STING pathway’s ability to restrict TLR activation, thereby limiting autoimmune manifestations in both experimental models (Motwani et al., 2021).

The cGAS-STING pathway drives SLE progression through multifaceted mechanisms spanning three biological hierarchies: (1) molecular-level DNA sensing and subsequent interferon production, (2) cellular-level dysregulation of immune cell activation, and (3) systemic-level amplification of autoimmune responses. Intriguingly, emerging evidence reveals context-dependent protective functions of this pathway, with its dualistic nature (pathogenic versus protective) highlighting the sophisticated regulatory complexity underlying SLE pathogenesis.

### 3.2 Systemic sclerosis

Systemic sclerosis (SSc) is an autoimmune disorder characterized by progressive fibrosis affecting both cutaneous and internal organs. The condition involves persistent autoimmune activation due to compromised immune regulation (Affandi et al., 2018). Within SSc pathogenesis, innate immunity plays a central role, where activated macrophages and dendritic cells release inflammatory factors including TNF-α and IL-6, amplifying inflammatory responses and promoting immune cell accumulation (York, 2011; Lescoat et al., 2021; Al-Adwi et al., 2023), eventually leading to tissue fibrosis. Advanced single-cell RNA sequencing studies have revealed intricate interactions among immune populations, fibroblasts, and endothelial cells during inflammation and aberrant tissue repair in SSc (Dai et al., 2022).

Disease lineage analysis from SSc revealed a significant shift in the activation pattern of the cGAS-STING pathway: from an “immune cell-led” activation pattern in SLE to a “stromal cell-immune cell crosstalk” pattern characteristic of SSc. This transformation highlights the unique mechanism of action of this pathway in tissue fibrosis. Paul S et al. identified strong associations between increased micronuclei presence and heightened expression of cGAS, IFN-β, and inflammatory markers in SSc. Their analysis of cGAS-STING pathway components showed enhanced levels and nuclear accumulation of phosphorylated IRF3 (Ser-396) within both limited (lcSSc) and diffuse (dcSSc) cutaneous SSc fibroblasts. Their work also documented increased concentrations of cGAS-synthesized 2′3′ cGAMP and elevated IFN-β production. Treatment with G150, a specific cGAS inhibitor, significantly decreased both 2′3′cGAMP activity and IFN-β synthesis in SSc fibroblasts (Paul et al., 2022), confirming the role of micronuclei in activating cGAS-STING signaling and subsequent IFN-β production (Paul et al., 2022). Further research by Jessica Bryon’s group showed that RNA within SSc fibroblast-derived extracellular vesicles enhanced type I IFN-induced STAT1 activation in keratinocytes, Ultimately promoting inflammatory responses and fibrotic processes (Bryon et al., 2024).

### 3.3 Psoriasis

Psoriasis represents a chronic inflammatory dermatological condition involving both adaptive and innate immune mechanisms. Its pathogenesis encompasses traditional immune cells (dendritic cells, macrophages, neutrophils) and non-conventional immune components (such as keratinocytes). Intercellular signaling occurs primarily through cytokine networks including TNF-α, IFN-γ, IL-17, and IL-22. These molecular mediators, combined with keratinocyte stimulation, trigger epidermal proliferation and induce the production of antimicrobial peptides, growth factors, and chemokines (Griffiths et al., 2021). This molecular cascade promotes characteristic psoriatic manifestations, including enhanced vascularity, neutrophilic infiltration, and proliferation of helper T cell type 1 (Th_1_) and type 17 (Th_17_) populations, establishing a self-perpetuating inflammatory loop (Hawkes et al., 2017).

Research has demonstrated that heightened cytoplasmic DNA levels contribute to psoriasis progression through STING-mediated mechanisms (Xu et al., 2024). Psoriatic lesions exhibit enhanced STING expression alongside upregulation of associated genetic components. Studies utilizing the imiquimod (IMQ)-induced psoriasis mouse model have established STING’s crucial function as an endogenous DNA detector. Experimental evidence shows that STING deficiency mitigates psoriatic manifestations and inflammation, while STING activation exacerbates these pathological changes, enhancing epidermal thickness and inflammatory responses (Pyclik et al., 2023).

Notably, the cGAS-STING pathway in psoriasis primarily functions through the epithelial-immune cell axis, amplifying inflammation via cross-talk between keratinocytes and immune cells. Yongsheng Y et al. demonstrated STING’s ability to activate NF-κB and enhance expression of inflammatory mediators including interleukin-1β, CCL20, and CXCL10 in both macrophages and keratinocytes, thereby amplifying psoriatic inflammation (Yu et al., 2022). Furthermore, various immune cells respond to DNA exposure by generating innate inflammatory responses. Dendritic cells detect DNA, generate type I interferons, and orchestrate initial psoriatic inflammation (Griffiths et al., 2021; Lou et al., 2020). Research by Xiaoyin S et al. utilizing DCs-specific STING knockout mice revealed suppressed DCs activation, resulting in decreased IL-17-producing T cells and Th1 populations, with subsequent reduction in IMQ-induced psoriatic inflammation (Sun et al., 2024).

### 3.4 STING-associated vasculopathy with onset in infancy (SAVI)

SAVI is a rare autoinflammatory disease caused by gain-of-function mutations in the STING gene. The disease is characterized by systemic inflammation, severe cutaneous vasculopathy, and interstitial lung disease, manifests in early infancy. The cutaneous manifestations typically present as erythematous to violaceous patches that may progress to necrotic gangrene, commonly affecting the cheeks, nose, ears, fingers, and toes (Munoz et al., 2015). Research has established that STING mutations can trigger type I interferonopathies presenting in early life, with a diverse spectrum of clinical presentations ranging from potentially fatal vascular disease to less severe chilblain lupus manifestations. Analysis shows that SAVI-associated STING variants can trigger spontaneous LBD rotation along the connecting helical loop or induce autonomous STING dimerization, even without the presence of the natural ligand 2′3′-cyclic guanosine monophosphate-adenosine monophosphate (2′3′-cGAMP), resulting in ligand-independent pathway activation (Liu et al., 2014). The most common mutations associated with SAVI include p. N154S and p. V155M (Dai et al., 2020). Keskitalo S et al. identified a previously undescribed gain-of-function mutation (G207E) in STING, characterized by a distinctive clinical profile including hair loss, light sensitivity, thyroid abnormalities, and SAVI-associated features such as livedo reticularis, vasculitis of the skin, nasal septum perforation, facial redness, and increased susceptibility to bacterial pathogens. Laboratory studies demonstrated that the G207E mutation led to constitutive activation of inflammatory pathways, resulting in dysregulated interferon signaling and enhanced inflammasome activity in peripheral blood mononuclear cells (Keskitalo et al., 2019).

## 4 Targeting the cGAS-STING pathway for treatment

The dysregulation of the cGAS-STING pathway can trigger a variety of inflammatory skin diseases, and modulating this pathway could potentially alleviate these conditions. In recent years, inhibitors targeting different sites within the cGAS-STING signaling pathway have been developed (Zhang S.-D. et al., 2024). This review highlights progress in developing cGAS and STING inhibitors for the treatment of inflammatory skin diseases (Table 1).

**TABLE 1:** Potential inhibitors targeting cGAS-STING for the treatment of inflammatory skin diseases.

Inhibitors	Targets	Molecular mechanisms	Clinical potential	Development stage
RU.521	cGAS	Inhibit cGAS mediated interferon production and reduce DNA-dependent type I interferon expression (Vincent et al., 2017)	SLE	Preclinical
Compound 25	cGAS	It showed significant anti-inflammatory properties in a mouse model of LPS-induced acute lung injury and may be useful for inflammatory skin diseases (Tan et al., 2021)	SLE	Preclinical
G150	cGAS	Directly bind the catalytic active site of cGAS, competitively block its binding to double-stranded DNA (dsDNA), thereby inhibiting the synthesis of the second messenger cGAMP (Lama et al., 2019)	SCC	Preclinical
H-151	STING	Covalently modifies STING’s C91 residues and inhibits TBK1 phosphorylation and type I interferon signaling, reducing systemic inflammation (Mukai et al., 2016; Haag et al., 2018)	Psoriasis	Preclinical-to-clinical transition
C-176	STING	Covalently modifies STING’s C91 residues and inhibits TBK1 phosphorylation and type I interferon signaling, reducing systemic inflammation (Mukai et al., 2016; Pan et al., 2021)	Psoriasis (species limitation)	Preclinical
ISD017	STING	Preventing STING transport from the endoplasmic reticulum to the Golgi apparatus, reducing inflammatory cytokines and type I interferon production (Li et al., 2024; Holm et al., 2012; Prabakaran et al., 2021)		Preclinical
Pt-CDs	cGAS-STING	Inhibit the secretion of proinflammatory cytokines by macrophages and restore the immune tolerance of keratinocytes (Petrasek et al., 2013)	Psoriasis	Proof-of-concept preclinical

### 4.1 cGAS inhibitor

RU.521, a compound with a well-defined structure-activity relationship, has been crystallographically characterized as a cGAS inhibitor bound to the enzyme’s active site in complex with double-stranded DNA (Vincent et al., 2017). It exhibits potency and selectivity in cellular assays involving cGAS-mediated signal transduction. Studies demonstrate that RU.521 potently inhibits cGAS-mediated interferon production while demonstrating minimal effects on cGAS-independent inflammatory cascades. The compound also shows remarkable efficacy in suppressing constitutive DNA-dependent type I interferon expression in primary macrophages derived from Trex1-deficient mice, which lack the ability to degrade cytoplasmic DNA (Vincent et al., 2017). Research by Lodoe Lama et al. introduced a series of compounds based on tetrahydro-1H-pyrido [4,3-b]indole (tetrahydro-γ-carboline, THγC) structure, showing varying degrees of inhibition against both human and mouse cGAS variants (Lama et al., 2019). However, these compounds demonstrated limited cellular penetration and failed to meet pharmaceutical development criteria for clinical investigation (Lama et al., 2019). Through structural refinement of the THγC scaffold, Jing Tan and associates developed Compound 25, a highly potent cGAS inhibitor. This molecule exhibited significant anti-inflammatory properties in a lipopolysaccharide (LPS)-induced acute lung injury mouse model (Tan et al., 2021) and shows potential therapeutic application for inflammatory skin conditions. However, substantial additional preclinical research remains necessary to validate these findings.

### 4.2 STING palmitoylation inhibitor

STING aggregation at the Golgi apparatus and subsequent recruitment of downstream signaling molecules is critically dependent on STING palmitoylation (Mukai et al., 2016). Research by Haag S M and colleagues revealed that H-151 inhibits palmitoylation-dependent STING clustering through covalent modification of STING’s C91 residue, thereby suppressing TBK1 phosphorylation and interferon-I signaling (Haag et al., 2018). This compound demonstrates efficacy in reducing IFN-I responses and systemic inflammation in *Trex1* ^−/−^ mice (Haag et al., 2018). Pan and associates discovered that topical H-151 application in psoriatic mouse models diminished skin pathology and markedly reduced pro-inflammatory cytokine production (IL-17, IL-23, IL-6), while decreasing M1 macrophage infiltration and Th17 cell development (Pan et al., 2021).

Although C-176 specifically targets murine STING (mSTING) rather than human STING (hSTING), it functions through covalent binding to Cys91, preventing activation-induced palmitoylation and subsequent formation of polymeric complexes at the Golgi apparatus, thus inhibiting downstream signaling cascades (Haag et al., 2018). Xiaoying S and colleagues investigated C-176s therapeutic potential in psoriasis treatment. Their findings showed that intraperitoneal C-176 administration significantly improved disease manifestations, including reduced ear swelling, decreased epidermal thickness and hyperplasia, and marked improvement in Psoriasis Area and Severity Index (PASI) scores (Sun et al., 2024). The research team also explored combination therapy using C-176 with anti-IL-17 A. Their analysis revealed that C-176 treatment reduced dendritic cell and γδ T cell populations within psoriatic lesions, and when combined with anti-IL-17A, exhibited enhanced anti-inflammatory effects (Sun et al., 2024). Ruolin L et al. reported that C-176 could mitigate NET-induced inflammation in THP-1 macrophages and inhibit HaCaT cell proliferation. In an *in vivo* psoriasis mouse model, C-176 dose-dependently improved skin lesions (Li et al., 2024).

### 4.3 STING transport inhibitor

Current research reveals that established cGAS and STING inhibitors, including H-151 and RU.521, demonstrate no inhibitory effects on STING activation through alternative pathways, such as those mediated by endoplasmic reticulum stress and extracellular vesicles (Holm et al., 2012). The STING pathway inhibitor ISD017 functions by preventing STING protein translocation from the endoplasmic reticulum to the Golgi apparatus, resulting in decreased inflammatory cytokine and type I interferon production, regardless of the upstream activation signal (Prabakaran et al., 2021; Petrasek et al., 2013). Research conducted by Alee I and colleagues showed that ISD017 administration in 24-week-old Fcgr2b-deficient mice enhanced survival rates and reduced glomerulonephritis progression. Their findings demonstrated that ISD017 treatment led to decreased populations of activated T cells (CD^4+^CD69^+^) and neutrophils (Ly6c^+^Ly6g^+^), while simultaneously reducing IL-1β expression and interferon-stimulated gene activity (Alee et al., 2024).

### 4.4 Nanoinhibitors

Nanoinhibitors, owing to their small size, often exhibit unique properties such as enhanced solubility, improved cellular uptake, and biofilm penetration capabilities (Albisa et al., 2017). They can be used in a variety of fields, including medicine, biotechnology and materials science. It has great potential to be applied widely because of its unique properties and its ability to selectively target and inhibit specific processes. Zhang et al. have designed positively charged Pt-CDs for their inhibition of the cGAS-STING pathway that restricts the secretion of pro-inflammatory cytokines from macrophages, restores the immune breakdown of keratinocyte immune tolerance, and overexpresses chemokines to break the positive loop of cytokines and break the homeostasis imbalance (Zhang Z. et al., 2024). We demonstrate that Pt-CDs locally applied have a therapeutic effect against psoriasis induced in an imiquimod mouse model with no significant toxicity (Zhang Z. et al., 2024).

## 5 Discussion

cGAS-STING pathway shows high sensitivity to cytoplasmic DNA in both the innate and adaptive immune processes and thus can be used to precisely detect cytoplasmic anomalies during both innate and adaptive immune pathways. Upon sensing abnormal DNA, cGAS catalyzes cGAMP production, which subsequently activates STING. This activation event is analogous to triggering a domino effect, initiating a series of signal cascades that are closely associated with the inflammatory response. These cascades precisely regulate the release of inflammatory cytokines and play a crucial regulatory role in maintaining the body’s immune balance.

The cGAS-STING pathway demonstrates remarkable functional versatility depending on context (Vincent et al., 2017). In physiological conditions, it serves a protective function in antimicrobial defense and tumor surveillance, facilitating swift recognition and elimination of pathogens and malignant cells (Bose, 2017). However, its hyperactivation in conditions like inflammatory skin disorders triggers pathological inflammatory cascades, resulting in tissue damage (Loo et al., 2020; Constanzo et al., 2021). The pathway’s regulation exhibits significant cell-type specificity. For instance, in dendritic cells, which function as immune system sentinels, cGAS-STING activation potentially modulates their antigen presentation capabilities and immune cell recruitment patterns (Li et al., 2019). Similarly, in T lymphocytes, which orchestrate immune responses, dysregulated cGAS-STING signaling can disrupt normal differentiation and activation processes, compromising immune homeostasis (Sun et al., 2024; Li et al., 2019; Zhang et al., 2021). Advancing our understanding of the cGAS-STING pathway’s contribution to inflammatory skin conditions necessitates deeper investigation into its disease-specific regulatory mechanisms. Particularly crucial is delineating its distinct functions within immune cells to facilitate the development of targeted therapeutic strategies.

Therapeutic development targeting the cGAS-STING pathway for inflammatory skin conditions has shown considerable advancement. Specific cGAS inhibitors, including RU.521 and Compound 25, effectively suppress inflammatory initiation through selective cGAS inhibition (Vincent et al., 2017; Tan et al., 2021), showing therapeutic promise in preliminary SLE studies. STING inhibitors, such as C-176 and H-151, demonstrate more diverse mechanisms of therapeutic action. These inhibitors can, respectively, inhibit the palmitoylation or polymerization process of STING, significantly alleviating the symptoms of inflammatory skin diseases such as psoriasis (Sun et al., 2024; Holm et al., 2012). However, the drug development process still faces numerous challenges. Ensuring target-specific action while minimizing interference with normal physiological processes (i.e., improving drug specificity) is crucial. Bioavailability is also a major issue. Effective drug delivery to the target site is essential. In addition, given the pathway’s role in immune defense, balancing inflammation suppression with maintaining basal immune function is critical to prevent increased susceptibility to pathogens due to over-inhibition. Emerging nanotechnology-based therapeutic approaches show significant promise in targeting cGAS-STING signaling. Specifically, platinum-doped carbon dots (Pt-CDs) demonstrate effectiveness in restoring immune homeostasis and reducing inflammatory mediator production through their enhanced biocompatibility and targeted delivery capabilities (Zhang Z. et al., 2024).

The exploration of combination therapeutic strategies represents a crucial avenue for future investigation. Research should focus on evaluating the coordinated application of cGAS-STING inhibitors with other immunomodulatory agents to enhance treatment efficacy. For example, simultaneous inhibition of cGAS-STING signaling to prevent inflammatory initiation and IL-17 blockade to suppress inflammatory mediator production offers improved inflammatory control through complementary mechanisms (Sun et al., 2024).

Recent years have witnessed substantial advances in understanding the cGAS-STING pathway’s role in inflammatory skin conditions. These investigations have enhanced our comprehension of disease mechanisms, while pathway-specific inhibitors have demonstrated therapeutic potential in preclinical studies and animal models. However, translating these laboratory findings into clinical applications remains challenging. The field’s future trajectory appears oriented toward integrating multi-omics approaches and precision medicine strategies. As research continues to advance the clinical applications of cGAS-STING pathway modulation, this therapeutic approach holds broad potential for treating inflammatory skin disorders.
